# Supplementary material for: Peel Diffusion and Antifungal Efficacy of Different Fungicides in Pear Fruit: Structure-Diffusion-Activity Relationships
Source: J Fungi (Basel). 2022 May 23;8(5):547. doi: 10.3390/jof8050547 (PMC9144380; doi:10.3390/jof8050547)
Supplement: Supplementary file 1 [file jof-08-00547-s001.zip › jof-1688174-supplementary.pdf]

Supplementary Information

Peel Diffusion and Antifungal Efficacy of Different Fungicides in Pear Fruit: Structure-Diffusion-Activity Relationships

Gui-Yang Zhu [1,†], Ying Chen [1,†], Su-Yan Wang [1], Xin-Chi Shi [1], Daniela D. Herrera-Balandrano [1], Victor Polo [2,*] and Pedro Laborda [1,*]

[1] School of Life Sciences, Nantong University, Nantong 226019, P. R. China

[2] Departamento de Química Física – Instituto de Biocomputación y Física de Sistemas Complejos (BIFI), Universidad de Zaragoza, Zaragoza 50009, Spain

[†] These authors contributed equally to this work.

[*] Correspondence to: P Laborda, School of Life Sciences, Nantong University, Nantong 226019, P. R. China. E-mail: pedro@ntu.edu.cn; V Polo, Departamento de Química Física – Instituto de Biocomputación y Física de Sistemas Complejos (BIFI), Universidad de Zaragoza, Zaragoza 50009, Spain. E-mail: vipolo@unizar.es

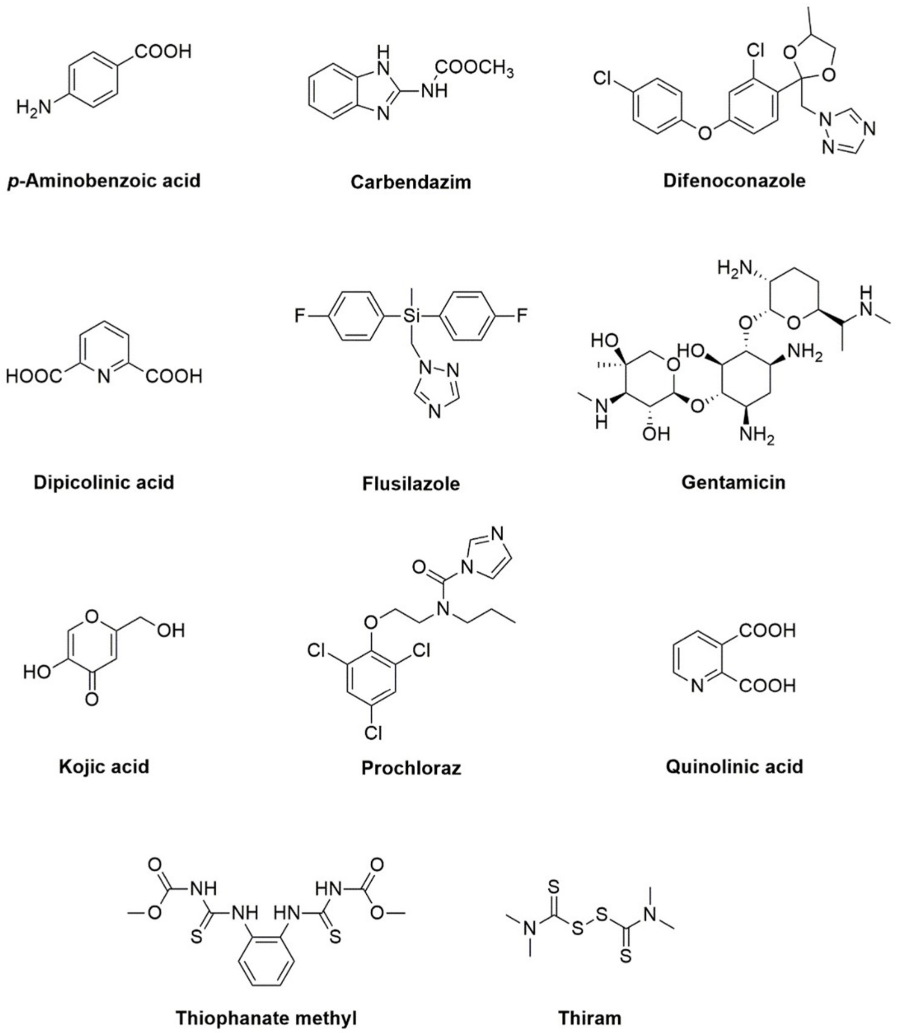

Figure S1. Structures of the studied fungicides.

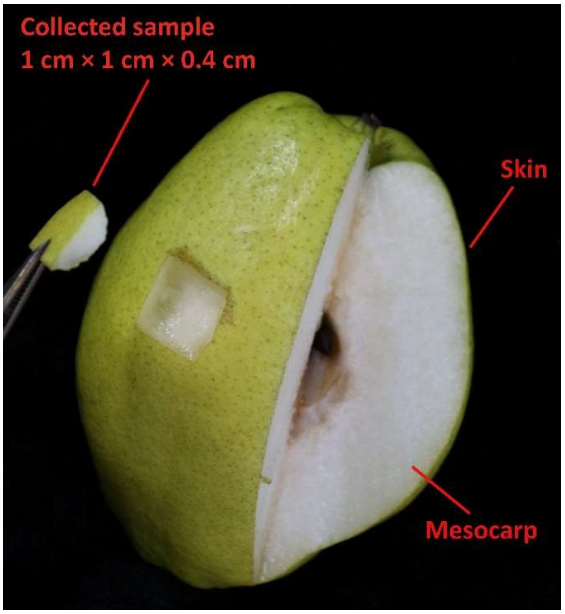

Figure S2. Extraction of samples from pears.

Table S1. DFT absolute energies (in a.u.) for the optimized structures calculated at the M062X(SMD)/6-311G(d,p) level.

**Compound**	**Energy**
p-Aminobenzoic acid	-476.12605
Carbendazim	-663.05413
Difenoconazole	-2044.41503
Dipicolinic acid	-625.38770
Flusilazole	-1272.05188
Gentamicin	-1586.97382
Kojic acid	-533.06635
Prochloraz	-2276.40178
Quinolinic acid	-625.38128
Thiophanate methyl	-1781.92663
Thiram	-1938.03024

Table S2. Cartesian coordinates (in a.u.) for all optimized structures (**A:** *p*-aminobenzoic acid; **B:** carbendazim; **C:** difenoconazole; **D:** dipicolinic acid; **E:** flusilazole; **F:** gentamicin; **G:** kojic acid; **H:** prochloraz; **I:** quinolinic acid; **J:** thiophanate methyl; and **K:** thiram).

A:

6	-2.107167	-0.036632	0.000043
6	-1.375504	-1.221596	0.000095
6	0.012703	-1.193388	0.000057
6	0.678874	0.030227	0.000005
6	-0.045564	1.221597	-0.000018
6	-1.430637	1.186021	-0.000015
1	-1.920784	-2.156943	0.000188
1	0.583467	-2.113041	0.000100
1	0.496173	2.159671	-0.000065
1	-2.001829	2.108264	-0.000065
6	2.162608	0.120445	-0.000025
8	2.787256	1.146535	0.000037
8	2.761423	-1.086064	-0.000072
1	3.712623	-0.919538	-0.000073
7	-3.541940	-0.128234	0.000066
1	-3.918743	0.348747	0.812838
1	-3.918643	0.346662	-0.813973

B:

6	4.187364	-0.312401	0.000047
6	3.170160	-1.253918	-0.000059
6	1.853097	-0.795586	-0.000058
6	1.596199	0.589404	0.000042
6	2.609246	1.540862	0.000144

6	3.912047	1.063763	0.000143
1	5.219015	-0.642724	0.000059
1	3.374311	-2.317109	-0.000123
1	2.394977	2.602364	0.000191
1	4.734255	1.768638	0.000228
1	-0.375625	1.511950	-0.000122
6	-0.253324	-0.586748	-0.000101
7	0.662418	-1.508560	-0.000099
7	0.215129	0.693180	0.000004
7	-1.608476	-0.880026	-0.000132
1	-1.845742	-1.861181	0.000903
6	-2.610316	0.049108	-0.000127
8	-2.451023	1.246480	-0.000443
8	-3.800183	-0.559540	0.000205
6	-4.924136	0.325121	0.000233
1	-4.908827	0.955649	-0.888510
1	-5.799191	-0.318220	0.000051
1	-4.909051	0.955324	0.889223

C:

6	6.194014	-0.623604	0.163517
6	6.177070	0.560583	-0.559212
6	5.059236	1.383040	-0.494916
6	3.981056	1.016080	0.297833
6	4.005182	-0.166076	1.030675
6	5.117117	-0.993201	0.959288
1	7.027946	0.832116	-1.170238
1	5.013113	2.312478	-1.047810
1	3.159524	-0.435172	1.652036

1	5.153496	-1.916719	1.522103
17	7.590613	-1.663353	0.073321
8	2.922927	1.894153	0.381523
6	1.644827	1.410878	0.285406
6	1.317706	0.329294	-0.524806
6	0.657506	2.084429	0.991925
6	-0.006741	-0.078541	-0.609223
1	2.080358	-0.192883	-1.087886
6	-0.658823	1.664462	0.882717
1	0.936563	2.927270	1.610803
6	-1.026033	0.577949	0.086979
1	-1.438020	2.180572	1.427616
17	-0.333665	-1.446238	-1.642244
6	-2.491088	0.140559	0.027900
6	-4.142805	1.726718	-0.369560
6	-3.480998	1.253471	-1.659382
1	-5.141863	1.285582	-0.276388
1	-4.176519	1.100259	-2.481894
1	-2.678836	1.938409	-1.965037
6	-2.713299	-1.176498	0.780505
1	-2.146850	-1.983537	0.320851
1	-2.394889	-1.040323	1.815412
6	-5.142957	-1.020571	1.426360
7	-4.564129	-2.465336	-0.131228
1	-5.010262	-0.255562	2.175465
6	-5.862150	-2.439779	0.080294
1	-6.537186	-3.078077	-0.467513
7	-6.274431	-1.562214	1.034554
7	-4.107727	-1.551035	0.745323

6	-4.187140	3.226329	-0.180751
1	-4.817740	3.684948	-0.945739
1	-4.599443	3.476818	0.797206
1	-3.180896	3.644552	-0.256020
8	-3.303557	1.132831	0.632247
8	-2.949706	-0.004575	-1.286911

D:

6	1.194091	1.544409	-0.000020
6	0.000005	2.250610	0.000017
6	-1.194071	1.544429	0.000044
6	-1.134755	0.150480	0.000036
6	1.134777	0.150483	-0.000049
1	2.152615	2.044412	-0.000036
1	0.000025	3.333581	0.000031
1	-2.152604	2.044415	0.000069
6	-2.391812	-0.679487	0.000012
6	2.391808	-0.679497	-0.000068
7	0.000000	-0.537314	-0.000013
8	-2.432278	-1.870728	-0.000028
8	2.432259	-1.870734	0.000086
8	-3.494808	0.098625	-0.000014
1	-4.254323	-0.498000	-0.000036
8	3.494792	0.098616	-0.000020
1	4.254310	-0.498005	0.000046

E:

14	-0.274547	-0.718501	0.577166
6	-0.075727	-1.382198	2.311700

1	-0.436235	-0.654463	3.042837
1	-0.649393	-2.304003	2.433336
1	0.970651	-1.609634	2.522540
6	0.362732	-1.992339	-0.702404
1	-0.072931	-2.984527	-0.562771
1	0.127942	-1.655499	-1.715153
6	-2.077801	-0.367195	0.192083
6	-2.769369	0.608700	0.924610
6	-2.785236	-1.064308	-0.793752
6	-4.108730	0.885652	0.685429
1	-2.253438	1.172335	1.696957
6	-4.128205	-0.806542	-1.048176
1	-2.292291	-1.832129	-1.381623
6	-4.764203	0.167032	-0.300690
1	-4.650157	1.638979	1.243470
1	-4.683137	-1.342127	-1.807879
6	0.758894	0.824628	0.318220
6	0.249542	1.973915	-0.298164
6	2.111698	0.822274	0.692531
6	1.050027	3.087383	-0.528909
1	-0.791204	2.006739	-0.604867
6	2.929684	1.920000	0.461361
1	2.545995	-0.054006	1.167066
6	2.377544	3.036207	-0.143768
1	0.666936	3.984297	-0.998824
1	3.977120	1.922869	0.735142
9	3.156696	4.104435	-0.365137
9	-6.057604	0.425471	-0.537145
7	1.810615	-2.112851	-0.612594

6	2.758818	-1.624420	-1.430288
6	3.678150	-2.504575	0.217388
1	2.526800	-1.111567	-2.351171
1	4.434920	-2.866572	0.895763
7	2.390805	-2.674043	0.463595
7	3.959057	-1.859694	-0.939234

F:

6	-3.851123	0.643976	-0.222525
6	-2.782228	-0.325537	0.287057
6	-4.344041	-1.941075	0.810121
6	-5.573055	-1.110308	0.409030
6	-5.120626	-0.103073	-0.665753
1	-2.425568	-0.952942	-0.545363
1	-3.436218	1.175797	-1.094373
1	-4.020740	-2.526442	-0.063014
1	-4.615724	-2.634437	1.610098
1	-4.877440	-0.712576	-1.552513
6	0.466354	-0.159491	0.019386
6	-0.710486	0.798582	-0.034697
6	-0.320486	2.209328	0.384612
6	0.865001	2.699083	-0.442138
6	2.079434	1.759749	-0.397772
6	1.626115	0.367349	-0.836528
1	-1.097552	0.839556	-1.062637
1	0.757763	-0.214410	1.076892
1	0.532250	2.805774	-1.482570
1	1.164141	3.696437	-0.102443
1	2.464937	1.692390	0.627381

1	1.256131	0.435963	-1.867851
1	-0.016837	2.149176	1.443161
8	-1.719807	0.367040	0.862448
6	3.274553	-1.025989	0.263736
6	3.684379	-2.472216	-0.009617
6	4.833486	-2.490793	-1.017368
6	5.964452	-1.580427	-0.536043
6	5.445765	-0.174494	-0.249795
1	5.187035	-3.515904	-1.161064
1	4.022532	-2.895733	0.940998
1	2.569166	-1.001450	1.096687
1	6.412859	-1.971339	0.381098
1	6.754167	-1.518074	-1.290286
1	5.089393	0.306515	-1.167792
1	4.449687	-2.131787	-1.978817
8	2.677833	-0.580795	-0.927128
8	-6.492201	-1.990252	-0.237228
1	-6.875408	-2.559821	0.436045
8	-4.145200	1.574296	0.798316
1	-5.041234	1.886327	0.610629
8	0.037395	-1.417605	-0.443042
1	0.731791	-2.071316	-0.253796
6	-6.196904	-0.462859	1.638479
1	-6.979115	0.243566	1.358190
1	-6.633433	-1.239627	2.275366
1	-5.430768	0.061527	2.208973
8	4.344809	-0.223159	0.670178
8	-3.293687	-1.144567	1.301138
7	2.473894	-3.186889	-0.431527

1	2.312447	-2.988151	-1.415488
1	2.600756	-4.188697	-0.342779
7	3.189116	2.196260	-1.243539
1	2.860818	2.302463	-2.200201
1	3.505832	3.112510	-0.942072
7	-1.480595	3.067783	0.170243
1	-2.270669	2.703254	0.701327
1	-1.290537	4.002047	0.516642
7	-6.117664	0.925287	-0.964475
1	-5.701530	1.551378	-1.648723
6	6.497609	0.713893	0.415151
1	7.381251	0.693022	-0.233365
6	6.008459	2.157951	0.535727
1	5.809465	2.586716	-0.448635
1	5.081771	2.184953	1.114882
1	6.758734	2.770790	1.042010
7	6.878249	0.108657	1.693627
1	7.555418	0.697975	2.165206
1	6.051521	0.072469	2.283510
6	-7.392467	0.447234	-1.501672
1	-7.269739	-0.267667	-2.325302
1	-7.964141	-0.051579	-0.721535
1	-7.962491	1.308707	-1.852593

G:

6	1.653815	-0.414409	0.000030
6	1.028581	0.919810	0.000007
6	-0.417799	0.899626	0.000029
6	-1.072618	-0.277399	0.000001

6	0.900791	-1.524379	-0.000026
1	-0.980369	1.821703	0.000068
1	1.279949	-2.534587	-0.000047
8	2.995499	-0.436490	0.000045
1	3.255398	0.500707	-0.000005
6	-2.561715	-0.446105	0.000054
1	-2.831676	-1.032911	0.887508
1	-2.831742	-1.033347	-0.887105
8	-3.155764	0.826454	-0.000235
1	-4.108450	0.718993	0.001214
8	1.749684	1.907336	-0.000022
8	-0.460600	-1.470227	-0.000063

H:

6	-4.474391	0.398774	-0.459639
6	-4.178778	-0.899049	-0.070604
6	-2.927834	-1.163410	0.466813
6	-1.977042	-0.156307	0.635752
6	-2.312921	1.134767	0.222371
6	-3.554192	1.427832	-0.320285
1	-4.904418	-1.692150	-0.185971
1	-3.796093	2.436762	-0.624754
17	-1.137629	2.395508	0.417015
17	-6.037904	0.744381	-1.136323
17	-2.527742	-2.777337	0.956652
8	-0.776422	-0.409531	1.208054
6	0.282534	-0.689759	0.289984
1	0.216609	-1.733483	-0.037554
1	0.201991	-0.044710	-0.590746

6	1.574666	-0.423647	1.043750
1	1.559325	-1.000633	1.973994
1	1.628384	0.629473	1.313775
7	2.735949	-0.818310	0.254390
6	3.805811	-0.009418	-0.004809
6	4.498402	2.339200	0.222630
6	2.432290	2.082351	-0.516047
6	2.778417	3.397254	-0.460147
1	1.545160	1.586079	-0.865593
1	2.180275	4.249310	-0.741906
1	5.474835	2.049211	0.577436
7	4.065856	3.546267	0.011521
7	3.543337	1.390545	-0.058589
8	4.925223	-0.413087	-0.201520
6	3.015822	-2.259085	0.170522
1	3.959456	-2.467556	0.682305
1	2.218340	-2.776933	0.708834
6	3.085201	-2.758954	-1.268266
1	2.132987	-2.548619	-1.765834
1	3.860899	-2.197883	-1.794427
6	3.393457	-4.252638	-1.318234
1	3.436842	-4.611014	-2.347339
1	4.357176	-4.465207	-0.849501
1	2.628840	-4.832069	-0.793846

I:

6	2.391538	1.233050	0.007660
6	2.763158	-0.106741	-0.024100
6	1.761946	-1.062211	-0.017745

6	0.432856	-0.650313	0.016963
6	0.169696	0.722900	0.022498
7	1.126094	1.645790	0.024572
1	1.976980	-2.124093	-0.032507
1	3.143465	2.015731	0.016475
1	3.808348	-0.386326	-0.046164
6	-1.224058	1.307853	-0.002114
6	-0.608907	-1.715968	0.075373
8	-1.705355	1.944998	0.883210
8	-0.399407	-2.869380	-0.176735
8	-1.821833	1.073203	-1.179619
1	-2.698575	1.478959	-1.136415
8	-1.800458	-1.257786	0.482491
1	-2.413829	-2.004508	0.480620

J:

6	-0.630824	3.291132	0.291409
6	0.631401	3.290800	-0.291353
6	1.262268	2.090013	-0.585607
6	0.639996	0.877080	-0.289739
6	-0.639960	0.877447	0.290994
6	-1.261965	2.090631	0.586247
1	-1.122966	4.225490	0.529060
1	1.123789	4.224897	-0.529531
1	2.232101	2.080378	-1.067473
1	-2.231820	2.081399	1.068084
7	1.226940	-0.370131	-0.595372
1	0.641449	-1.053274	-1.058675
7	-1.227319	-0.369473	0.597276

1	-0.642351	-1.052270	1.061736
6	2.540054	-0.762468	-0.501587
6	-2.540455	-0.761563	0.502907
16	3.048421	-2.140588	-1.251100
16	-3.049548	-2.139264	1.252819
7	3.281939	0.075422	0.306166
1	2.772926	0.731921	0.885756
7	-3.281712	0.076198	-0.305524
1	-2.772210	0.732174	-0.885289
6	4.648754	0.132627	0.595380
6	-4.648210	0.132664	-0.596535
8	-5.048546	0.840408	-1.480573
8	5.050043	0.841472	1.478110
8	-5.390888	-0.611256	0.197772
8	5.390642	-0.611979	-0.199041
6	-6.791580	-0.587848	-0.094943
1	-6.967401	-0.927218	-1.115371
1	-7.242588	-1.267749	0.621498
1	-7.184957	0.421582	0.023257
6	6.791746	-0.587443	0.091568
1	7.242079	-1.268022	-0.624660
1	7.184441	0.422024	-0.028504
1	6.969241	-0.925395	1.112184

K:

7	-3.304807	0.481779	0.000400
6	-2.184844	-0.263122	-0.000276
6	-4.597015	-0.198936	0.000955
1	-4.689905	-0.833995	-0.881823

1	-4.688187	-0.835684	0.882678
1	-5.383475	0.551944	0.002465
6	-3.263778	1.941940	0.001125
1	-4.281463	2.321817	0.000157
1	-2.751481	2.317558	0.891197
1	-2.749722	2.318296	-0.887609
16	-0.716474	0.765787	-0.000365
16	0.716478	-0.765682	-0.000396
16	-2.171912	-1.923742	-0.001022
6	2.184833	0.263124	-0.000118
6	3.263604	-1.941992	0.001396
1	2.749574	-2.317385	0.890558
1	2.751187	-2.318485	-0.888240
1	4.281240	-2.322001	0.002560
6	4.597064	0.198746	0.000469
1	4.689370	0.834106	-0.882148
1	4.688992	0.835178	0.882349
1	5.383448	-0.552217	0.001090
7	3.304779	-0.481830	0.000600
16	2.171997	1.923803	-0.000813

A

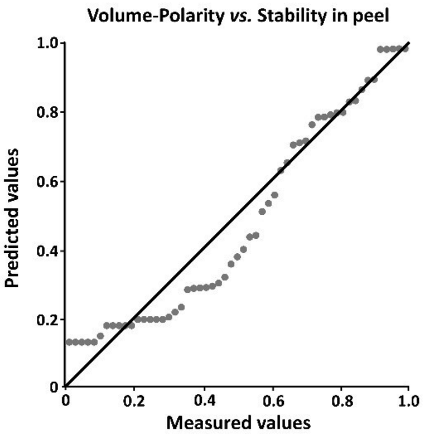

B

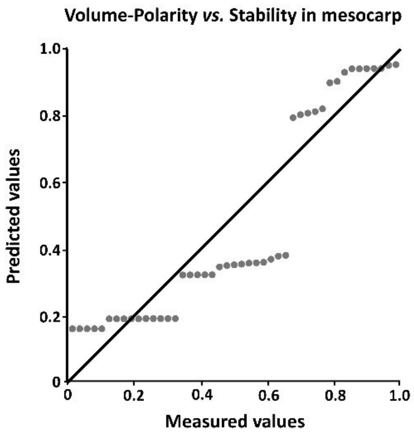

Figure S3. Correlations between (**A**) volume, polarity and stability in pear peel, and (**B**) volume, polarity and stability in pear mesocarp. Linear regression analyses were performed using SPSS software version 16.0.
